# Phosphorus starvation induces the synthesis of novel lipid class diacylglyceryl glucuronide and diacylglyceryl‐*N,N,N*‐trimethylhomoserine in two species of cold‐adapted microalgae *Raphidonema* (Chlorophyta)

**DOI:** 10.1111/tpj.17227

**Published:** 2025-01-27

**Authors:** Hirono Suzuki, Stéphan Cuiné, Bertrand Légeret, René H. Wijffels, Chris J. Hulatt, Yonghua Li‐Beisson, Viswanath Kiron

**Affiliations:** ^1^ Faculty of Biosciences and Aquaculture Nord University Bodø Norway; ^2^ Aix Marseille Univ, CEA, CNRS, BIAM, Institut de Biosciences et Biotechnologies Aix‐Marseille, CEA Cadarache Saint Paul‐Lez‐Durance France; ^3^ Bioprocess Engineering, AlgaePARC Wageningen University Wageningen the Netherlands

**Keywords:** extremophile, lipid remodeling, membrane lipid, phosphorus starvation, triacylglycerol

## Abstract

Microalgae possess diverse lipid classes as components of structural membranes and have adopted various lipid remodeling strategies involving phospholipids to cope with a phosphorus (P)‐limited environment. Here, we report a unique adaptative strategy to P deficient conditions in two cold‐adapted microalgae, *Raphidonema monicae* and *Raphidonema nivale,* involving the lipid class diacylglyceryl glucuronide (DGGA) and the betaine lipid diacylglyceryl‐*N,N,N*‐trimethylhomoserine. Lipidomic analyses showed that these two lipid classes were present only in trace amounts in nutrient replete conditions, whereas they significantly increased under P‐starvation concomitant with a reduction in phospholipids, suggesting a physiological significance of these lipid classes to combat P‐starvation. Additionally, we found two putative sulfoquinovosyldiacylglycerol (SQDG) synthases, known to be involved in DGGA synthesis in higher plants, in the draft genome of *R. monicae*, and compared it with SQDG synthases found in other organisms such as higher plants, Streptophyta, and Chlorophyta. DGGA has not been previously recognized in Chlorophyta, and our findings suggest that the lipid class may be present in other closely related green algae too. Thus, this study expands our knowledge on diverse lipid remodeling responses of Chlorophycean algae to adapt to low P environments.

## Significance Statement

Membrane lipid remodeling is a strategy that microalgae and plants have employed to combat phosphorus limiting environments, playing an important role in preserving metabolic performance. Here we demonstrated a novel lipid remodeling strategy in cold‐adapted microalgae *Raphidonema* (Chlorophyta) involving diacylglyceryl glucuronide and diacylglyceryl‐*N,N,N*‐trimethylhomoserine, which significantly contribute to phospholipid substitution. The discovery expands our understanding of diverse lipid remodeling responses adopted by microalgae, particularly those living in extreme environments.

## INTRODUCTION

Microalgal lipids are valuable compounds with growing applications in animal and human nutrition (Borowitzka, 2013). Most microalgal lipids are glycerolipids that consist of glycerol backbones with at least one fatty acid molecule bound to the *sn*‐1 or *sn*‐2 positions, plus a third fatty acid, a phosphorus group, a sugar molecule or a betaine moiety at position *sn*‐3. In this range of configurations, algal lipids comprise hundreds of different molecular species that belong to diverse lipid classes, with various functions ranging from structural roles, such as constituting photosynthetic membranes, to energy storage and cell signaling processes. Different types of lipids are therefore localized to specific organelles and cellular structures. For example, the storage lipid triacylglycerol (TAG) can accumulate in cytoplasmic lipid droplets, whilst the galactolipids monogalactosyldiacylglycerol (MGDG) and digalactosyldiacylglycerol (DGDG), and the sulfolipid sulfoquinovosyldiacylglycerol (SQDG) are exclusively found in the chloroplast. Other classes such as betaine lipids and phospholipids constitute the non‐chloroplast membranes (i.e., mitochondrial, endoplasmic reticulum, ER, and plasma membranes), whilst phosphatidylglycerol (PG) can be found in both thylakoid membranes and extra‐chloroplast membranes. Due to the diverse evolutionary history of plastid‐bearing microalgae, the composition of glycerolipid classes and lipid molecular species varies amongst different taxonomic groups and can be substantially remodeled when cells are exposed to stress conditions (Cañavate et al., 2017a; Cañavate et al., 2017b; Li‐Beisson et al., 2019).

Phosphorus (P) is often a limiting nutrient in aquatic ecosystems, and microalgae have acquired various strategies to cope with P‐limited environments. One such strategy is cell membrane lipid remodeling, whereby the composition of lipid classes is altered to preserve metabolic performance (Cañavate et al., 2017a; Van Mooy et al., 2009). For example, during P‐starvation, microalgae may replace the phospholipid PG with the sulfolipid SQDG (Martin et al., 2010; Van Mooy et al., 2009; Yu et al., 2002). Additionally, DGDG can serve as a substitute for phospholipids in P‐deficient environments and has been observed to be exported from the chloroplast to extra‐chloroplast membranes in higher plants (Jouhet et al., 2004). In the diatom *Thalassiosira pseudonana* (Thalassiosiraceae), phosphatidylcholine (PC) can even be substituted with diglycosylceramide (Hunter et al., 2018). Furthermore, betaine lipids have demonstrated roles in cell membrane homeostasis under P‐starvation in various microalgal species (Van Mooy et al., 2009), including diacylglyceryl‐*N,N,N*‐trimethylhomoserine (DGTS) in the Eustigmatophyceae *Nannochrolopsis oceania* (Murakami et al., 2018), and DGTS and diacylglycerylcarboxyhydroxymethylcholine (DGCC) in the Prymnesiophyceae *Emiliania huxleyi* (Haptophyta) (Shemi et al., 2016). These lipids increase in abundance at the expense of PC or both PC and PG, respectively. Thus, microalgae exhibit significant metabolic flexibility and adaptive responses across taxonomic groups with respect to their functional lipid species.

Despite the diversity and abundance of glycerolipids in microalgae, the lipidomic architecture of many algal lineages and their responses to physiological stress have barely been explored. Particularly, the lipid composition of Trebouxiophyceae (Chlorophyta), one of the most ubiquitous and diverse group within Chlorophyta that inhabit both aquatic and terrestrial environments has only been reported for a few species including *Chlorella* sp. (Lu et al., 2013), *Picochlorum* sp. (Cañavate et al., 2017b), and *Lobosphaera incisa* (Bigogno et al., 2002). The lipid composition of Trebouxiophyceae differs amongst strains, and there is little evidence for the effect of P‐starvation on lipids in this ecologically successful group. The genus *Raphidonema* is typically found in cold environments including snow and glaciers in polar and alpine regions (Segawa et al., 2023; Yakimovich et al., 2021). In snow and ice habitats microorganisms experience multiple forms of stress including variable light intensities, cold temperatures (Hoham & Remias, 2020; Leya et al., 2009), and nutrient concentrations that fluctuate and are often very low (Darcy et al., 2018; Morgan‐Kiss et al., 2006). Long chain‐polyunsaturated fatty acids (LC‐PUFAs) are believed to be beneficial to low temperature adaptation (Morgan‐Kiss et al., 2006) and are present in *Raphidonema* (Suzuki et al., 2019). The lipid class DGTS is shown to be important for cold adaptation in *Nannochloropsis* (Murakami et al., 2018), but has not been extensively studied in other microalgae. We hypothesized that *Raphidonema* may have acquired ability to replace phospholipids with non‐P containing lipids such as DGTS under P‐limited conditions through selective pressures in cold habitats.

In this work, *Raphidonema monicae* (Prasiolales, Trebouxiophyceae, Yakimovich et al., 2021) and *Raphidonema nivale* were studied as representatives of cold‐adapted microalgae from the underexplored order Prasiolales. We first characterized their lipid class and molecular species and then investigated the lipid species dynamics upon P‐starvation. This study offers detailed lipidomic information of two cold‐adapted microalgae *R. monicae* and *R. nivale*, and we further provide insights into physiological and biochemical mechanisms for the adaptation to P‐deficient environment.

## RESULTS

### Effect of P‐starvation on growth, cellular P content, and fatty acid composition

**Figure 1:**
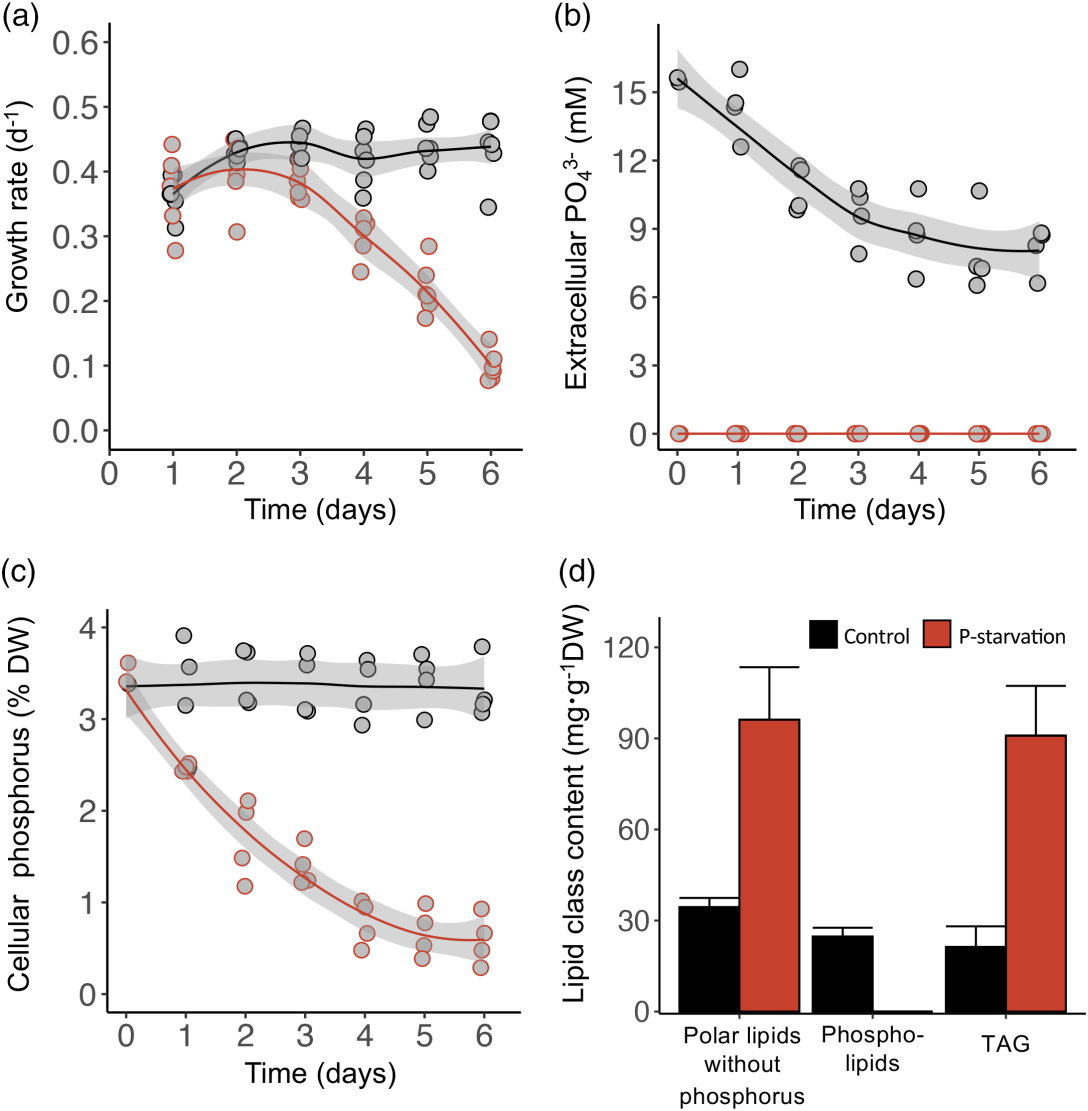
Physiological parameters and lipid class compositions of *Raphidonema monicae* over 6 days in turbidostat cultures. (a) Growth rate (d^−1^), (b) extracellular PO_4_^3−^ concentration (mM), (c) cellular phosphorus content (%DW), (d) lipid class content (mg·g^−1^DW) at day 6. DW, dry weight; TAG, triacylglycerol. Black color in each graph represents control and red color represents P‐starved cells. Data are the means and error bars represent the standard deviations of four biological replicates.

**Figure 2:**
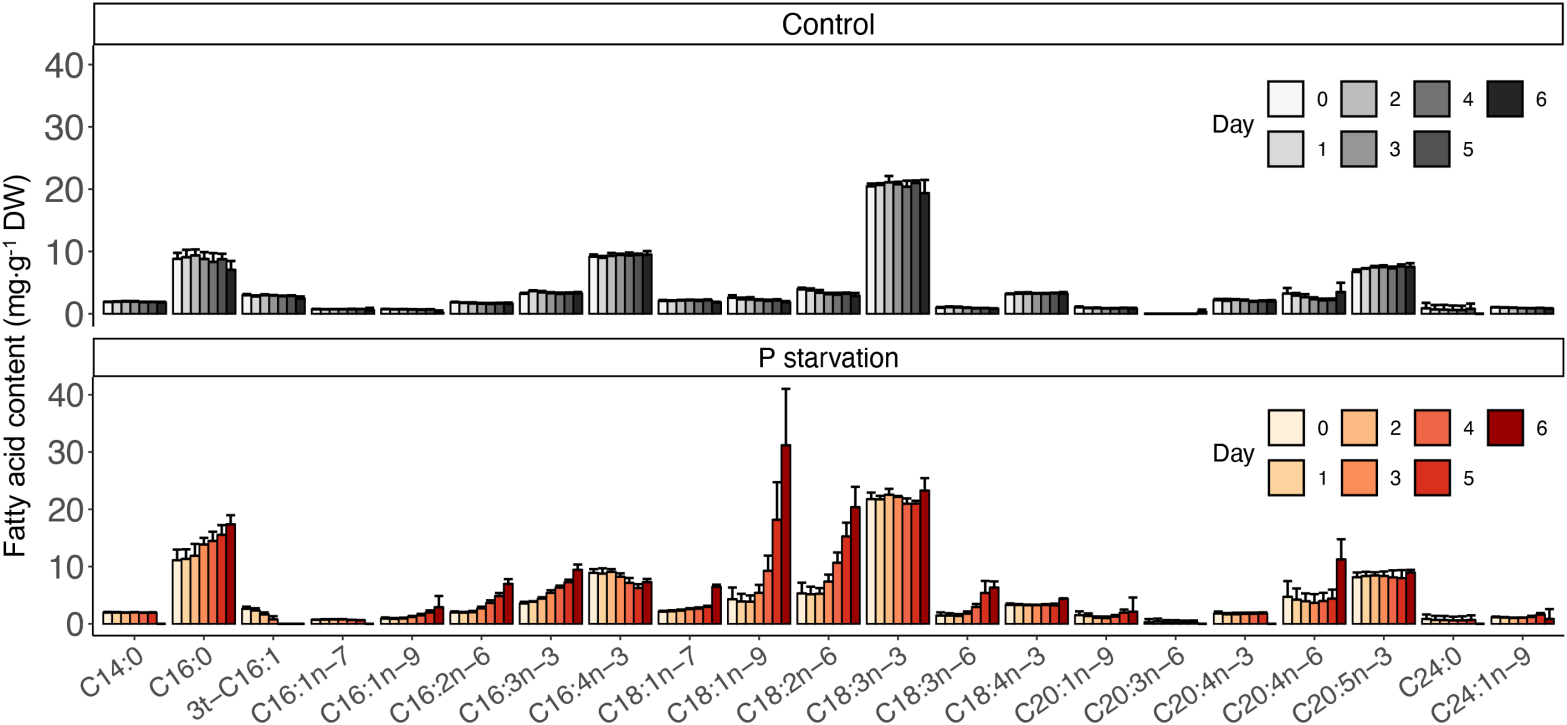
Fatty acid content (mg·g^−1^ DW) of *Raphidonema monicae* over 6 days in turbidostat cultures. DW, dry weight; 3 t‐C16:1, 3‐trans‐hexadecenoic acid. Data are the means and error bars represent the standard deviations of four biological replicates.

*Raphidonema monicae* was cultivated in both nutrient‐replete (control) and P‐starved conditions with turbidostat mode in flat‐plate photobioreactors to determine the effect of P starvation on growth, cellular P content, fatty acids, and lipid class composition. There was a substantial difference in growth between the treatments. Control cultures exhibited steady‐state growth at a mean specific growth rate of 0.42 ± 0.01 d^−1^ (Figure 1a), whereas the growth rate of P‐starved cultures was 0.38 ± 0.02 d^−1^ at day 3 and decreased substantially to 0.10 ± 0.02 d^−1^ at day 6. Measurements of extracellular PO_4_^3−^ and total cellular P concentrations confirmed that control cultures were not limited by P in the medium (Figure 1b), and that total cellular phosphorus remained approximately constant at 3.37 ± 0.06 %DW throughout (Figure 1c). The cellular total P content of P‐starved cultures decreased from 3.31% ± 0.24% DW at the start of the treatment to 0.63% ± 0.26% DW at day 6 of P‐starvation (Figure 1c). Phospholipids, polar lipids without P, and TAG were also measured in control and P‐starved cells at day 6 (Figure 1d). In the control treatment, phospholipids accounted for 24.7 ± 2.9 mg⋅g^−1^ DW, but they were undetectable in P‐starved cells. Polar lipids without P in control cultures amounted to 34.4 ± 3.0 mg⋅g^−1^ DW, whereas those of P‐starved cells were much higher, accounting for 96.2 ± 17.3 mg⋅g^−1^ DW. The 6 days of P‐starved cultivation produced the cells containing 90.9 ± 16.4 mg⋅g^−1^ DW of TAG, higher than controls that contained only 21.3 ± 6.8 mg⋅g^−1^ DW TAG. The main fatty acids observed in *R. monicae* were C16:0, C16:4n‐3, and C18:3n‐3; these remained consistent over 6 days in control conditions (Figure 2). Under P‐starvation, fatty acids C16:0, C16:1n‐9, C16:2n‐6, C16:3n‐3, C18:1n‐9, C18:2n‐6, and C18:3n‐6 each increased, whilst others did not change significantly (Figure 2). 3 t‐C16:1 (3‐trans‐hexadecenoic acid) was present in control treatments but disappeared on day 6.

### Characterization of glycerolipidome of *Raphidonema monicae*

**Figure 3:**
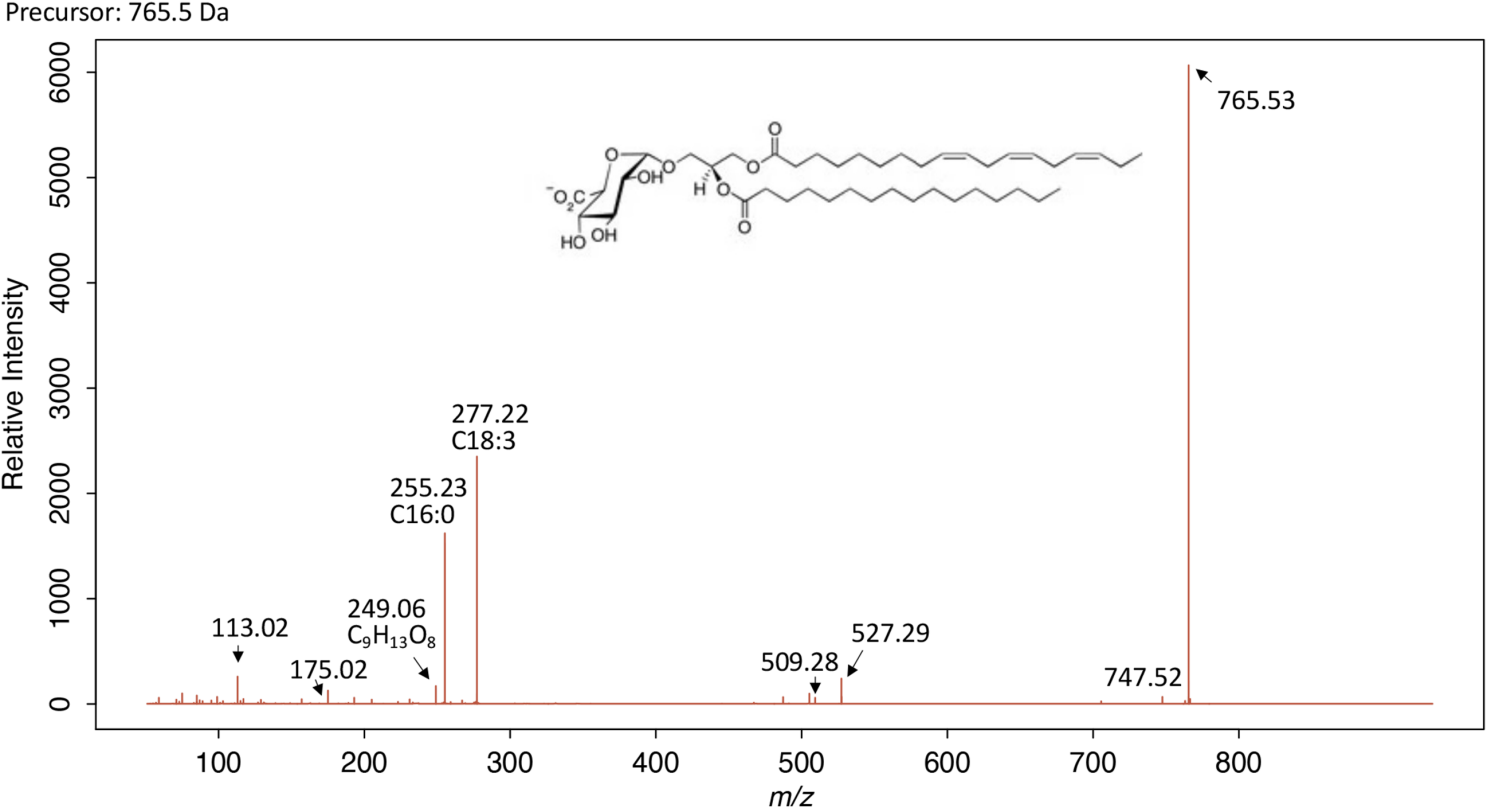
Mass spectrum of diacylglyceryl glucuronide in *Raphidonema monicae* analyzed by ultra‐performance liquid chromatography equipped with a mass spectrometry. The number next to the peaks or indicated by arrow represent *m/z* of each peak. The major molecule *m/z* 765.53 exhibits the three signals (*m/z* 249.06, 255.23, and 277.22) with two signals for the loss of C18:3 and C16:0 (*m/z* 509.3, and 527.3).

To identify the glycerolipids present in *R. monicae*, we analyzed both control (nutrient replete) and P‐starved cells using one‐dimensional high performance thin‐layer chromatography (HPTLC) run together with lipid class standards. In nutrient‐replete conditions, the lipid composition of *R. monicae* revealed the occurrence of classic chloroplastidic lipids (MGDG, DGDG, SQDG, and PG), three major classes of phospholipids (PC, PI, and PE), and neutral lipids (TAG and DAG). Additionally, the betaine lipid DGTS and an unknown spot were present in significant quantities in P‐starved conditions, but found only in traces or were not quantifiable under P‐replete conditions. The unidentified spots were subsequently separated by two dimensional HPTLC and recovered from the plate, and further analyzed by UPLC‐MS/MS. This revealed the mass spectra of the product—ion spectrum of *m*/*z* 766 exhibiting three signals (*m*/*z* 249.06, 255.23, and 277.22); leading to its identification as diacylglyceryl glucuronide (DGGA), with two signals for the loss of C18:3 and C16:0 (*m*/*z* 509.3 and 527.3) (Figure 3). The fragmentation features were similar to that of DGGA identified in *A. thaliana* (Okazaki et al., 2013).

The lipid molecular species of *R. monicae* were analyzed by UPLC‐MS/MS (Table S2). The two galactoglycolipid classes MGDG and DGDG were characterized by molecular species that contained a combination of C16 and C18 polyunsaturated fatty acids. SQDG had C16:0 and C18:3 as major fatty acids, whilst PG mainly contained C16:0 or C16:1. VLC‐PUFAs (carbon chain length ≥ C20) were absent from the chloroplast membrane lipids MGDG, DGDG, PG, and SQDG, and confined to the extra‐chloroplastidic lipids PE, PC, and DGTS, of which the di‐VLC‐PUFA combinations including C20:4/C20:4 and C20:4/C20:5 were observed only in PC and PE. Short chain C16 and C18 series were widespread species across lipid classes, whereas C20:5 and C20:4 were primarily found in PE and PC.

### Glycerolipid composition in *Raphidonema monicae* and in closely related species *Raphidonema nivale*

**Figure 4:**
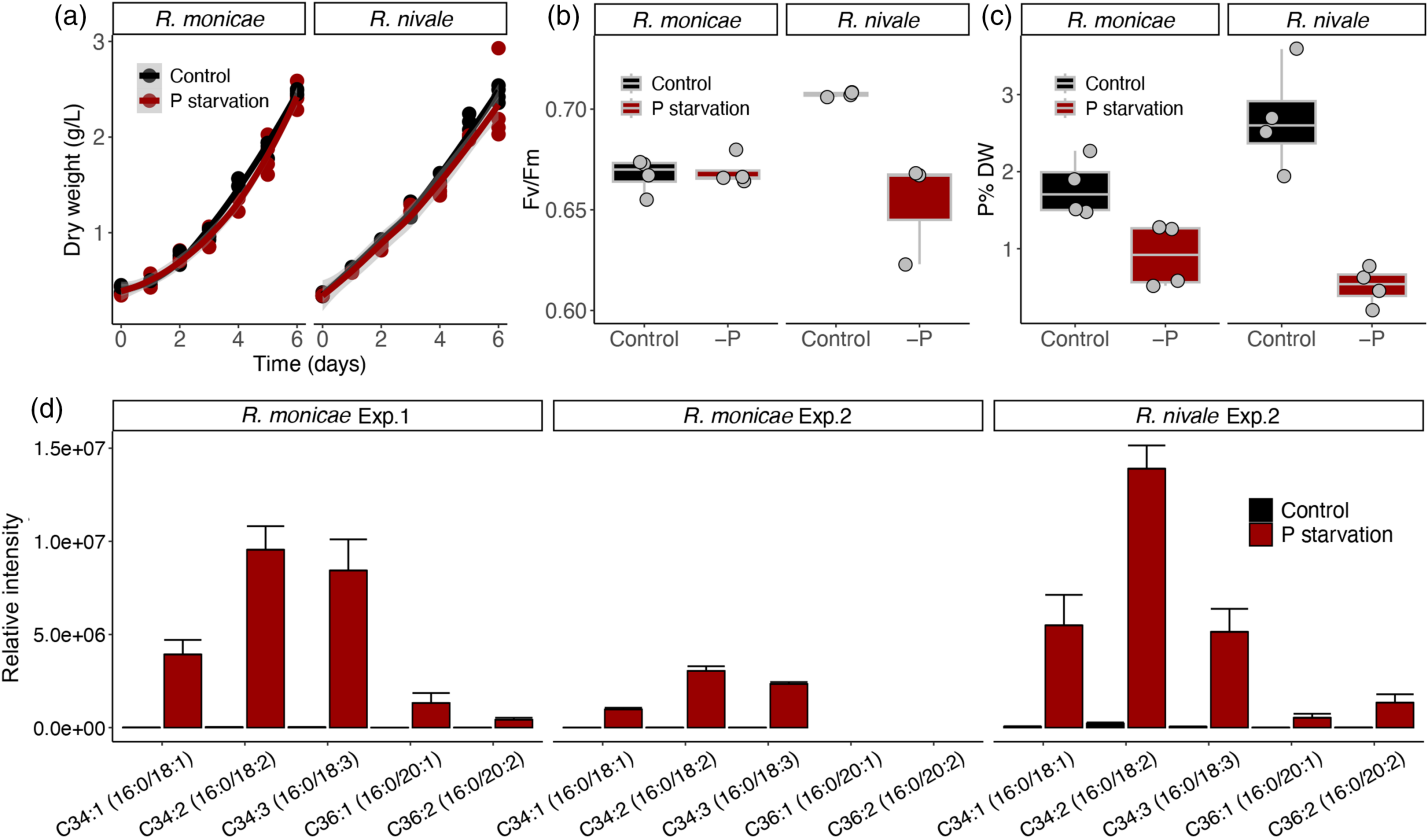
Physiological parameters and lipid class compositions of *Raphidonema monicae* and *Raphidonema nivale*. (a) Growth, (b) maximum quantum yield of photosystem II (Fv/Fm) and (c) cellular phosphorus content (P% DW) in control (non‐P starved) and P‐starved at day 6. (d) the lipid molecular species of diacylglyceryl glucuronide in control and P‐starved at day 6 in *R. monicae* in turbidostat (Experiment 1; left) and batch cultures (Experiment 2; middle), and *R. nivale* in batch cultures (Experiment 2; right). Data are the means and error bars represent the standard deviations of four biological replicates.

**Figure 5:**
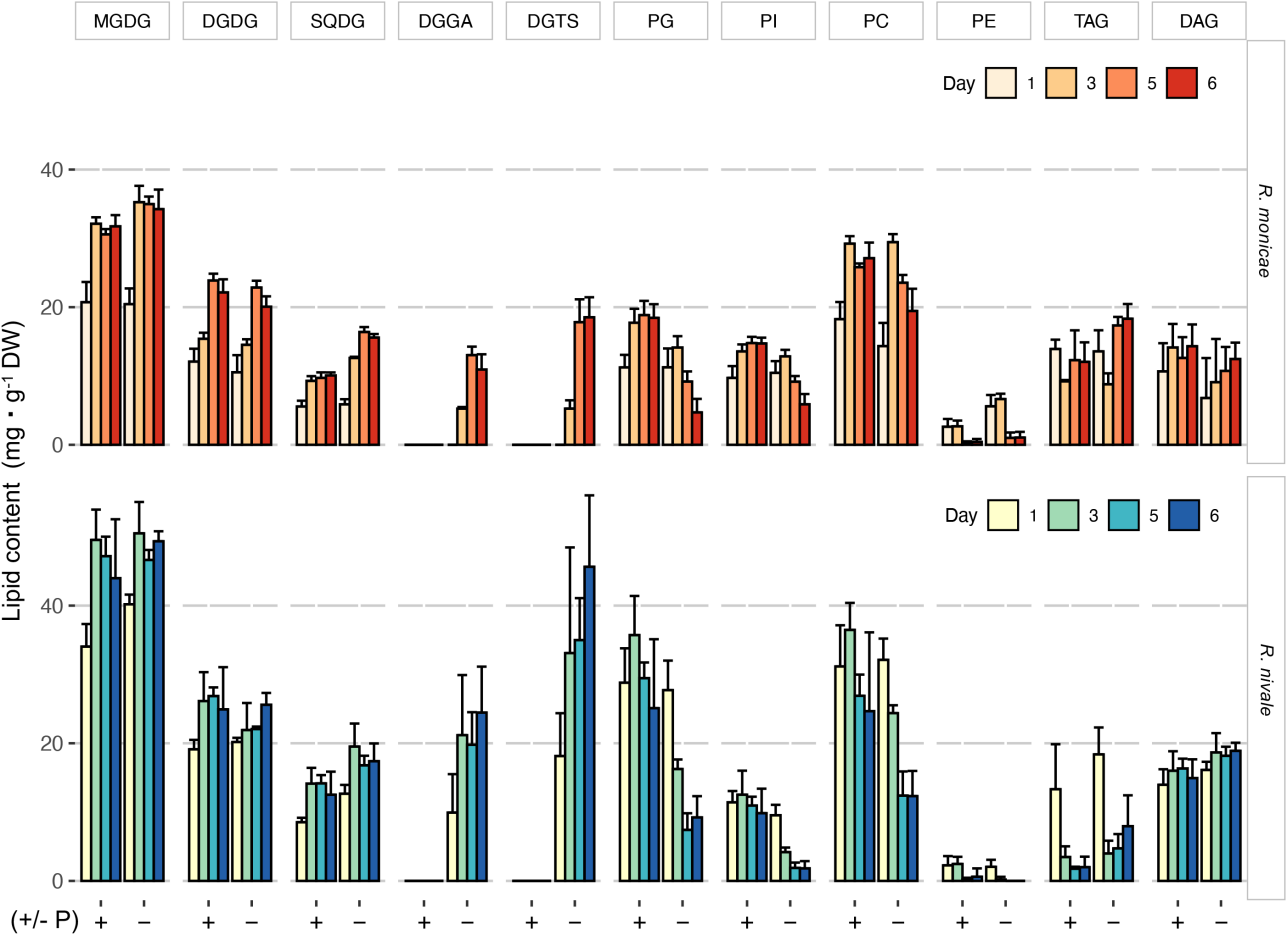
Lipid class dynamics of *Raphidonema monicae* and *Raphidonema nivale* over 6 days in control and P‐starved cultures in batch cultures. Data are the means and error bars represent the standard deviations of four biological replicates. DAG, diacylglycerol; DGDG, digalactosyldiacylglycerol; DGGA, diacylglyceryl glucuronide; DGTS, 1,2‐diacylglyceryl‐3‐*O*‐4′‐(*N,N,N*‐trimethyl)‐homoserine; MGDG, monogalactosyldiacylglycerol; PC, phosphatidylcholine; PE, phosphatidylethanolamine; PG, phosphatidylglycerol; PI, phosphatidylinositol; SQDG, sulfoquinovosyldiacylglycerol; TAG, triacylglycerol.

To assess whether the unusual lipid DGGA is present more widely in the genus, *R. monicae* and *Raphidonema nivale* ARK‐S02‐19 were cultivated over 6 days in photobioreactors in batch cultures and their phenotypes and glycerolipid class profiles were compared. The growth of *R. monicae* and *R. nivale* was comparable both in control and P‐starved treatments (Figure 4a). The Fv/Fm of *R. monicae* was not significantly different between the treatments but was reduced in *R. nivale* exposed to P‐removal from the medium for 6 days (Figure 4b). The cellular P content of P‐starved cells of *R. monicae* at day 6 amounted to 0.91 ± 0.42 %DW, which was half that of the controls, whereas that of P‐starved cells of *R. nivale* amounted to 0.51 ± 0.25 %DW, one fifth of control cells (Figure 4c). The DGGA species consisted of C16:0/18:1, C16:0/18:2, and C16:0/18:3 in P‐starved cells of *R. monicae* in the batch cultures, though C16:0/20:1 and C16:0/20:2 were also present in *R. monicae* on day 6 of P‐starvation in turbidostat cultures and P‐starved *R. nivale* in batch cultures (Figure 4d). Analyses of *R. nivale* grown both in control and P‐starved conditions revealed that they shared the same lipid classes with *R. monicae*, though in slightly different proportions (Figure 5). As observed in *R. monicae*, *R. nivale* also contained significant amounts of the lipid class DGGA in P‐starved cells.

### Membrane lipid remodeling dynamics under P‐starvation in *Raphidonema* species

**Figure 6:**
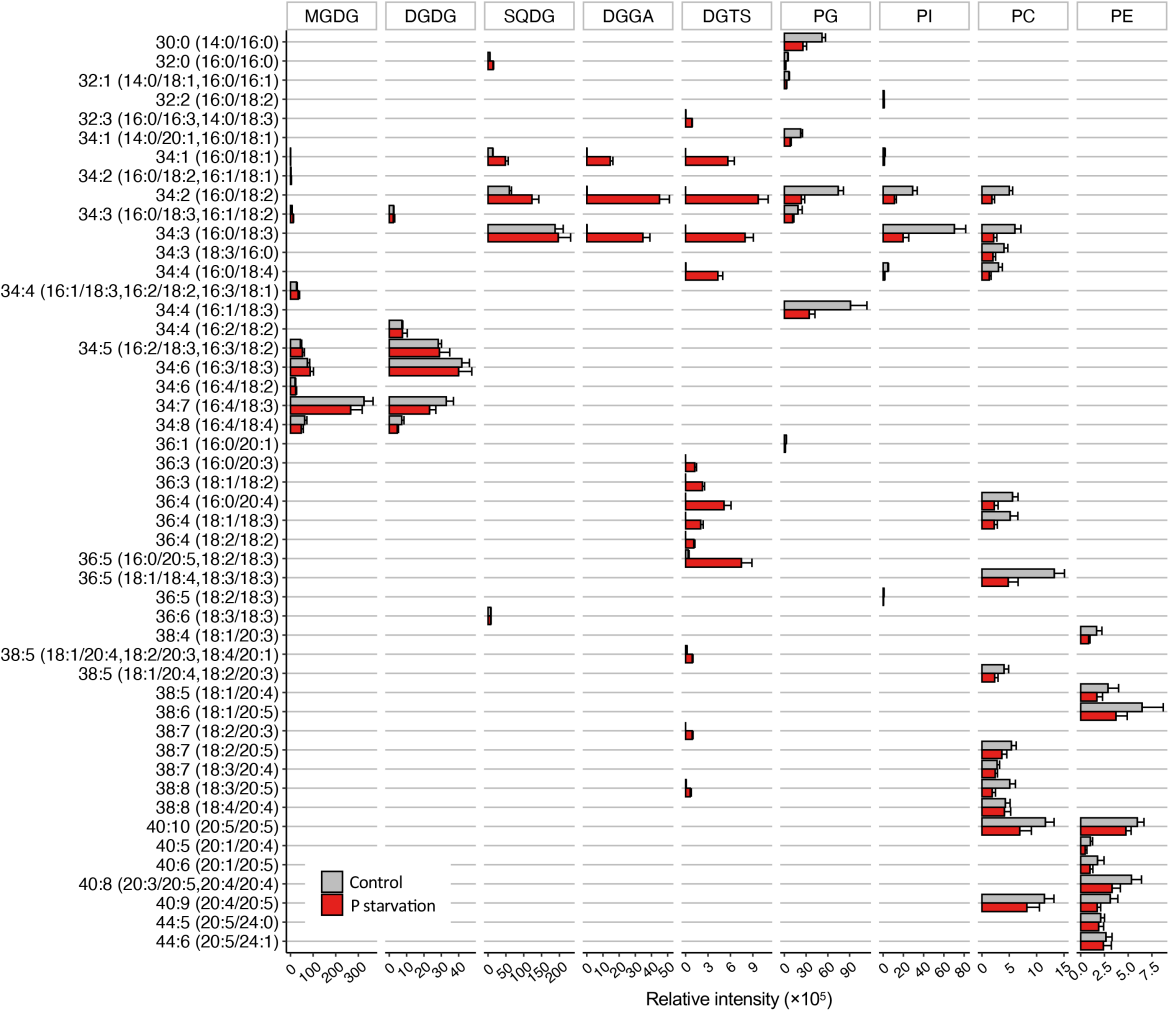
Relative abundances of lipid molecular species across all lipid classes in *Raphidonema monicae* at day 6 in control (gray) and P‐starved (red) conditions in batch cultures. The fatty acid combinations are shown, and their sn‐positions were not identified. Two fatty acid combinations were quantified and presented together when peaks were not separated. Data represent the mean ± standard deviation of four biological replicates. DGDG, digalactosyldiacylglycerol; DGGA, diacylglyceryl glucuronide; DGTS, 1,2‐diacylglyceryl‐3‐*O*‐4′‐(*N,N,N*‐trimethyl)‐homoserine; PC, phosphatidylcholine; PE, phosphatidylethanolamine; PG, phosphatidylglycerol; PI, phosphatidylinositol; MGDG, monogalactosyldiacylglycerol;; SQDG, sulfoquinovosyldiacylglycerol.

To investigate the effect of P‐starvation on lipid composition, we measured the dynamics of lipid classes and lipid molecular species in *R. monicae* and *R. nivale* from the batch culture experiments (Figures 5, 6; Figures S2–S5). Under P‐starvation, both species maintained the abundance of chloroplast membrane lipids MGDG and DGDG whilst all phospholipids including PG, PI, PE, and PC decreased (Figure 5). The amounts of SQDG in both species were slightly higher in P‐starved cells compared with controls at day 6. Both DGTS and DGGA were absent in control treatments but increased significantly in response to P‐starvation. The kinetics of DGTS and DGGA accumulation differed slightly between species, where these lipid classes appeared from day 1 in *R. nivale* but from day 3 in *R. monicae*. The amount of DGGA was 2.2 times higher in *R. nivale* than in *R. monicae* on day 6, whilst PG was 2.5 times higher in *R. nivale* than in *R. monicae* in control treatments. Similarly, the DGTS content was 2.5 times higher in *R. nivale* than in *R. monicae* on day 6. Besides, the relative change of lipid molecular species under P‐starvation at day 6 was also measured in *R. monicae* (Figure 6). DGTS had 14 molecular species compared to DGGA that contained only three molecular species. DGTS has molecular species that are more similar to PC than to other lipid classes and contains the intermediate fatty acids that appear during desaturation and/or elongation steps of C18 and C20 fatty acids towards highly unsaturated fatty acids. The amount of each lipid molecular species of both DGTS and DGGA increased in response to P‐starvation (Figure 6; Figures S4, S5). On the other hand, the majority of PC lipid molecular species exhibited reduced quantities, with varying degrees of downregulation in P‐starved conditions compared with control cultures. Likewise, under P‐starved conditions, the PE molecular species that contained VLC‐PUFAs displayed a wide range of reductions compared to the controls. The most substantial reductions were observed in C38:6 (18:1/20:5) with a 42.6% decrease, and in C40:8 (20:4/20:5, 20:4/20:4), which decreased by 38.2%. P‐starvation also caused a pronounced decline in the major lipid molecular species of PI and PG. Amongst PI molecular species, C34:3 (16:0/18:3) and C34:2 (16:0/18:2) exhibited the largest reductions, decreasing by 72.0% and 61.9%, respectively. For PG lipid molecular species, C34:4 (16:1/18:3) and C34:2 (16:0/18:2) showed the most significant reductions, with decreases of 62.4% and 53.3%, respectively.

### Identification of putative SQDG synthase homologs that are required for DGGA synthesis in *R. monicae*

**Figure 7:**
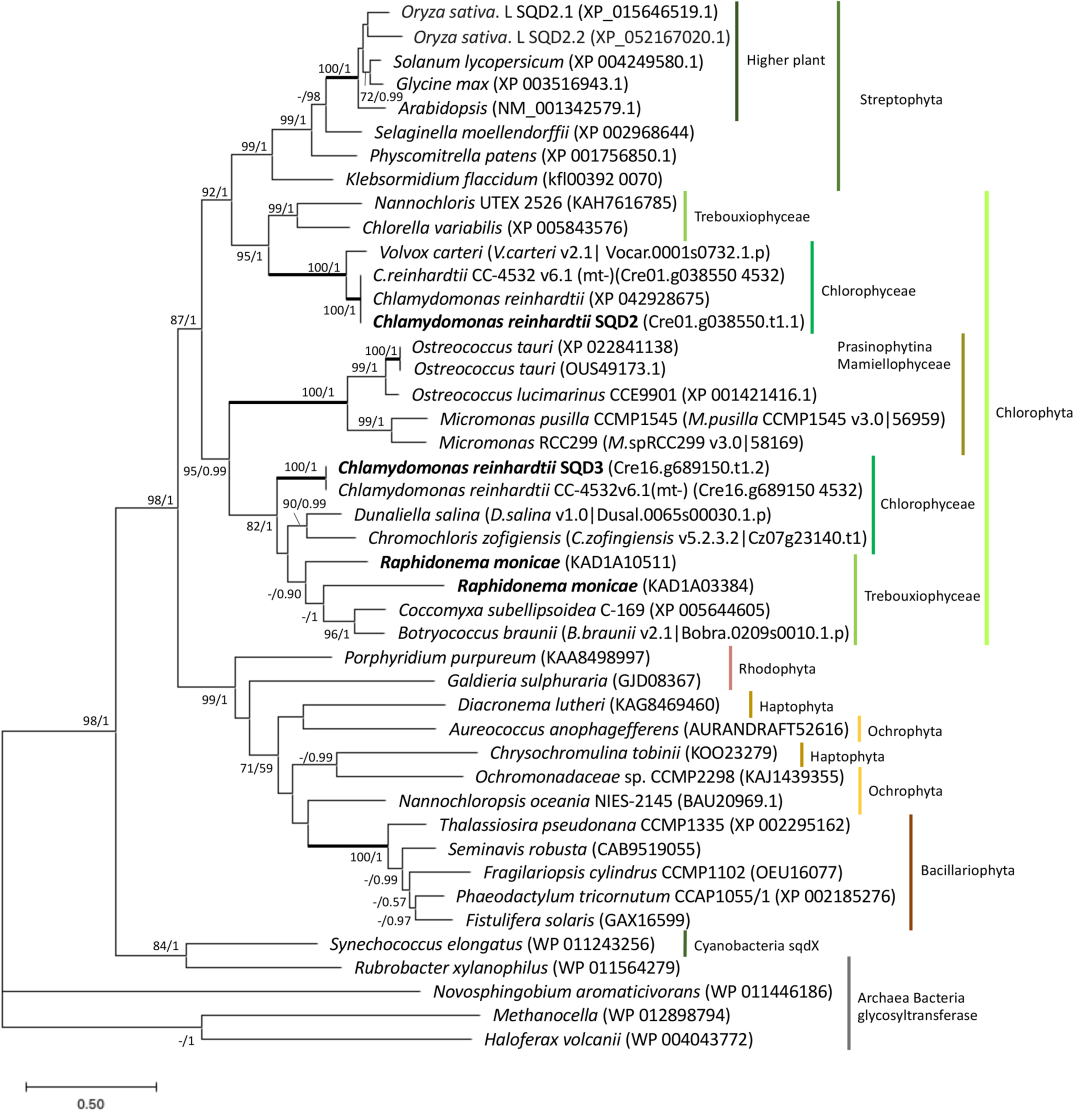
Maximum likelihood phylogenetic tree of sulfoquinovosyldiacylglycerol synthase based on amino acid sequences in Streptophyta, Chlorophyta, and algae from red algae lineage. Archaea and bacteria glycosyltransferase serve as the outgroup. Our new sequences (KAD1A10511 and KAD1A03384) in *Raphidonema monicae* and *Chlamydomonas reinhardtii SQD2* and *SQD3* are shown in bold. Numbers next to branches indicate statistical support values (maximum likelihood bootstraps (1000 replicates)/Bayesian posterior probabilities). The bootstrap support values above 70% and Bayesian posterior probabilities above 0.90 are shown. Thick lines indicate the branches with full statistical support (ML/BI:100/1.00).

In the land plant *Arabidopsis*, SQDG synthase (*SQD2* or *SQD3*) is required for DGGA synthesis (Okazaki et al., 2013). Therefore, we searched and detected two putative genes of SQDG synthase within the draft genome of *R. monicae* based on their protein sequence homology with 273 proteins reported in marine eukaryotes (Riccio et al., 2020). The phylogenetic analyses of amino acid sequences of these genes in *R. monicae* and the annotated genes across various species showed that the SQDG synthase of the red algae lineage (eukaryotes containing primary and secondary red algae‐derived plastids) is clearly separated from those of the green algae lineage (eukaryotes containing primary green plastids) (Figure 7). The cluster of green algae lineage was divided into two groups: a clade consisting of Streptophyta, Trebouxiophyceae and Chlorophyceae (Chlorophyta), and a clade comprising Mamiellophyceae, Trebouxiophyceae and Chlorophyceae (Chlorophyta). The two putative genes of SQDG synthase in *R. monicae* are both located in the same clade as *SQD3* from *C. reinhardtii*, rather than another sister clade containing *SQD2* from *C. reinhardtii* and *A. thaliana*. The SQDG synthases in *R. monicae* clustered together with those of related Trebouxiophycean species *Coccomyxa* and *Botryococcus*, although additional Trebouxiophyceae species *Nannochloris* and *Chlorella variabilis* were also found in a further, separate clade. SQDG synthases were present in a wide range of taxonomic groups in primary plastid‐bearing green lineages including Streptophyta, Trebouxiophyceae, Chlorophyceae, and Mamiellophyceae.

## DISCUSSION

Phosphorus is a central component of phospholipids, yet inorganic P is often a limiting nutrient in aquatic systems. The supply of P therefore plays a key role in glycerolipid metabolism and shapes the adaptive responses of microalgae in both natural ecosystems and industrial bioprocesses. This study revealed the presence of the lipid class DGGA in the Chlorophyta and illustrates the impact of P‐starvation more widely on the cellular glycerolipid metabolism and physiology of two *Raphidonema* species, which are adapted to low‐temperature environments.

### Glycerolipid profile and lipid remodeling of *Raphidonema monicae*

During optimal growth, *R. monicae* contained the typical chloroplast membrane lipids MGDG, DGDG, SQDG and PG, and that of extra‐chloroplast membrane lipids, that is, PE, PI, PC, and trace amount of DGTS. Very long chain polyunsaturated fatty acids (VLC‐PUFAs, carbon chain length ≥ C20) was confined to extra‐chloroplast membrane lipids, where di‐VLC‐PUFA combinations were observed only in PC and PE. Chloroplast membrane lipids exclusively contained fatty acids with carbon chains up to C18. As expected, all phospholipid classes PE, PI, PC, and PG decreased under P‐starvation. Interestingly DGTS and DGGA were not present or present in only trace amounts under P‐replete conditions, but substantially synthesized under P‐deficiency; the two findings are discussed further in the sections below.

### DGTS biosynthesis under P‐starvation

The betaine lipid DGTS is distributed across evolutionally diverse protist groups, and are often present in the Chlorophyta, Ochrophyta and Haptophyta (Cañavate et al., 2016; Cañavate et al., 2017a). From a biophysiochemical perspective, DGTS and PC are both bilayer forming lipids that share a similar zwitterionic structures (Sato & Murata, 1991) and are widely believed to be functionally interchangeable (Hoffmann & Shachar‐Hill, 2023). PC is typically found in extra‐chloroplast membranes (Hoffmann & Shachar‐Hill, 2023), whilst knowledge on the cellular localization of DGTS in microalgae is limited (Janero & Barrnett, 1982; Künzler et al., 1997; Sheffer et al., 1986). Microalgae with high amounts of DGTS often do not contain PC, or contain PC in very low amounts (Cañavate et al., 2017a), for example *Dunaliella* and *Chlamydomonas reinhardtii* (Chlorophyceae, Chlorophyta). Studies of the effect of P‐starvation on the DGTS content is limited to non‐PC‐containing *C. reinhardtii*, where DGTS accounted for approximately 26% of total fatty acids in nutrient replete conditions and remained stable in response to P‐starvation (Riekhof et al., 2003). In contrast, DGTS is only a minor lipid component in Trebouxiophyceae (Chlorophyta) such as *Chlorella* sp. (Cecchin et al., 2019), *Lobosphaera incisa* (Bigogno et al., 2002), and *Picochloroum* (Cañavate et al., 2017a) that contain DGTS ranging from 1.3% to 6.1% of total lipids (Bigogno et al., 2002; Cañavate et al., 2016; Weber et al., 1989), but known to increase under P‐starvation in *Picochlorum* and *Lobosphaera* (Cañavate et al., 2017a; Kokabi et al., 2020). Although *Chlorella kessleri* and *Chlorella pyrenoidosa* were previously found to be exceptions lacking DGTS amongst Trebouxiophyceae (Martin et al., 2014), a recent study showed that *Chlorella kessleri* actively synthesizes DGTS only under P‐starvation (Oishi et al., 2022). Here, our study also confirmed the same trend in *R. monicae* and *R. nivale* that synthesize DGTS in substantial amounts only under P‐starved conditions. DGTS in *R. monicae* was mainly composed of diverse lipid molecular species; C16:0 or C18 and C20 unsaturated fatty acids. Very long chain fatty acids (VLC‐FAs, carbon chain length ≥ C20) are synthesized via the elongation of acyl‐CoA or substrate fatty acids through fatty acids elongation complexes, which exclusively occurs in endoplasmic reticulum in higher plants (Chapman & Ohlrogge, 2012), fungi, and microalgae (Ruiz‐Lopez et al., 2015; Venegas‐Calerón et al., 2010). The presence of VLC‐FAs in DGTS in *R. monicae* suggests that the lipid class is most likely to be synthesized from DAG via the eukaryotic pathway, where the fatty acids are assembled into glycerolipids in the endoplasmic reticulum. Indeed, *R. monicae* seems to have clear distinctions in fatty acid composition between chloroplast or extra‐chloroplast lipids where VLC‐FAs are confined only to extra‐chloroplast lipids including PC, PE, PG, and DGTS. In *R. monicae*, DGTS and PC shared similar acyl chain composition and appeared to compensate for each other, depending on the P availability. Under P‐starvation, the accumulation of DGTS molecular species was accompanied by reduction in PC molecular species with the same acyl chain combinations including C34:1, C34:2, C34:3, C34:4, C36:4, and C36:5 (Figure 6). On the contrary, the zwitterionic non‐bilayer forming lipids PE were enriched in C20 PUFAs and did not contain any of the same molecular species as in DGTS. Given that C20 PUFAs are known to increase membrane fluidity (Vásquez et al., 2014, Baccouch et al., 2023), high inclusion of such lipids in PE suggests this lipid class may not functionally or structurally replace DGTS. Altogether, we propose that DGTS may substitute for phospholipids PC, and possibly PE for the construction of extra‐chloroplast membranes in *R. monicae* under P‐starvation. It is worth noting that the pronounced increase of DGTS also could be attributed to roles as an acyl donor during increased fatty acid synthesis as observed by Eichenberger and Gribi (1997) and/or as a component of monolayer lipid‐coat of lipid droplets (Goold et al., 2016).

While PC is thought to be substituted by DGTS in some microalgae, the extent to which it could functionally replace the former remains to be explored. PC acts as a major site of extra‐chloroplast lipid desaturation and distributor of PUFAs into other lipids by diacylation or re‐acylation cycles, referred to as acyl editing (Hoffmann & Shachar‐Hill, 2023). In *C. reinhardtii*, which does not possess PC, DGTS is known to serve as the acyl carrier in the desaturation of oleic and linoleic acids, whilst in *Nannochloropsis oceanica* it was involved in a similar manner for C18 fatty acid desaturation, besides its involvement in EPA biosynthesis (Meng et al., 2019). In *R. monicae*, DGTS contained a variety of intermediates involved in the desaturation and/or elongation C18 and C20 fatty acids, leading to the formation of highly unsaturated species such as C38:8 (18:3/20:5). These intermediates increased under P‐starvation, implying that DGTS may act as hub of C18 fatty acid desaturation and their subsequent elongation to C20 fatty acids, potentially compensating for the loss of PC under these conditions. However, specific di‐VLC‐PUFA species such as C40:9 (20:4/20:5) and C40:10 (20:5/20:5), which were detected in PC, were absent in DGTS. This absence suggests that DGTS may not be able to host di‐VLC‐PUFA species, limiting its ability to fully replace PC although further investigation is required to elucidate the underlying reasons for these observed differences. The dynamics of lipid remodeling between PC and DGTS in VLC‐PUFA producing green algae remain poorly understood. *Raphidonema* may therefore serve as an attractive model for studying these interactions under P‐starvation, particularly in relation to VLC‐PUFA biosynthesis and their biophysiochemical properties.

### DGGA biosynthesis under P‐starvation

Our findings indicate that P‐starvation induces the synthesis of another lipid class DGGA in *R. monicae* and *R. nivale*, suggesting the physiological significance of this lipid molecule. DGGA has been rarely reported in unicellular algae, including in some Haptophyta such as *Diacronema lutheri* (Eichenberger & Gribi, 1997; Lowenstein et al., 2021), *Chrysotila pseudoroscoffensis* (Moreira et al., 2022), and Ochrophyta such as *Ochromonas danica* (Ochrophyta, Eichenberger & Gribi, 1994). It has also been observed in higher plants, including *Arabidopsis*, rice, soybeans, and tomato (Okazaki et al., 2013; Okazaki et al., 2015; Okazaki et al., 2017). DGGA accounted for up to 1.9% and 3% of total glycerolipids in non‐P‐limiting conditions in *D. lutheri* and *O. danica* respectively (Eichenberger & Gribi, 1994; Eichenberger & Gribi, 1997). In higher plants, DGGA is typically present in low amounts under optimal conditions and is substantially synthesized under P‐starvation (Okazaki et al., 2013, Okazaki et al., 2015, Okazaki et al., 2017).

In the model plant *A. thaliana*, SQDG synthase (*SQD2*) is required for DGGA synthesis where it catalyzes the final step of SQDG synthesis by converting uridine‐diphospho‐sulfoquinovose (UDP‐SQ) and DAG into SQDG (Okazaki et al., 2013). Experimental confirmation shows that growth of *sqd2* mutants of *A. thaliana* that lack both SQDG and DGGA under P‐limited conditions, was severely impaired compared to other mutants lacking a gene catalyzing a reaction at earlier steps of the SQDG synthesis such as *sqd1* and *ugp3‐1*, leading to the absence of only SQDG as phenotype (Okazaki et al., 2013). The SQDG synthases of higher plants and microalgae are known to be orthologs of the cyanobacterial *sqdX* genes, which are distinct and unrelated to the *sqdD* gene encoding SQDG synthase in the alpha‐proteobacteria *Rhodobacter sphaeroides* and *Sinorhizobium melilti* (Benning et al., 2008; Güler et al., 2000; Rossak et al., 1995), and are present in many algal taxonomic groups (Riccio et al., 2020). In this study, we searched for the gene in the newly established genome of *R. monicae* and identified two SQDG synthases. Phylogenetic analyses of amino acid sequences of SQDG synthase showed that the SQDG synthases are present in a wide range of taxonomic groups in primary plastid‐bearing green lineages including Streptophyta, Trebouxiophyceae, Chlorophyceae, and Mamiellophyceae, suggesting their potential ability to synthesize DGGA. In the Trebouxiophyceae two clades of SQDG synthase‐encoding genes are observed, with both those of *R. monicae* clustered with those in Chlorophyceae that include the *SQD3* homolog of *C. reinhardtii*, and adjacent to those of Mamiellophyceae. Considering the substantial diversity of related Trebouxiophycean and Chlorophycean algae, including those that have adapted to extreme environments, DGGA synthesis may be more widespread amongst Chlorophyta. The functional difference between the SQDG synthase homologs forming different clades remain unknown, although the mRNA expression levels of *SQD2* and *SQD3* in *C. reinhardtii* have been reported to differ under various stress conditions (Bajhaiya et al., 2016; Boyle et al., 2012; Choi et al., 2021; Légeret et al., 2016). Further studies on the gene expression in response to abiotic stressors, or generating SQDG synthase knockout mutants in *Raphidonema monicae* and other relevant algae, will help to identify potential functional differences between *SQD2* and *SQD3* genes and provide insight into the function and roles of the SQDG synthases in relation to DGGA synthesis.

Okazaki et al. (2013) previously suggested that DGGA is synthesized by catalysis of DAG and UDP‐glucuronic acid by *SQD2* and this reaction potentially competes with SQDG synthesis based on the similarity of the acyl moieties of SQDG and DGGA in *A. thaliana*. Our lipidomic analyses of *R. monicae* have also shown that SQDG and DGGA share comparable acyl moieties composed primarily of C34:1 (16:0/C18:1), C34:2 (16:0/18:2), and C34:3 (16:0/18:3), suggesting a possible association of these lipid classes in their biosynthetic pathways in *R. monicae*. In higher plants, DGGA biosynthesis is proposed to take place in the chloroplast due to the localization of *SQD2* to the inner envelope of the chloroplasts (Okazaki et al., 2013). Interestingly, C36:1 (16:0/20:1) and C36:2 (16:0/20:2) were also present in DGGA of *R. monicae* and *R. nivale* where there was a greater reduction in cellular P content. The presence of C20:1, which is likely synthesized in the endoplasmic reticulum, suggests that DGGA is either exported to extra‐chloroplast membranes after synthesis by *SQD2* as in the case for DGDG in higher plants (Härtel et al., 2000), or alternatively synthesized in the ER through pathways that are yet to be described.

Amongst the four phospholipid classes that were reduced under P‐starvation, PG and PI are the only anionic bilayer‐forming lipids with comparable properties to DGGA. PG and PI in *Raphidonema* have a similar acyl chain profile to DGGA, and they maintained quantitatively inverse relationships with DGGA under P‐starvation. SQDG is known to replace PG under P‐starvation to maintain an anionic lipid environment in thylakoid membranes (Bolik et al., 2022). Although the inverse relationships between SQDG and PG were observed in *R. monicae* and *R. nivale* in the present study, DGGA may still have roles in replacing PG under P‐starvation, based on their similarities in acyl chain profile and regulations. For example, C20:1 was exclusively found in PG as C34:1 (14:0/20:1) or C36:1 (16:0/20:1) and DGGA as C36:1 (16:0/20:1) in *R. monicae*. Substantial differences in DGGA and PG content between the species have been also observed where the contents were 2.2 and 2.6 times higher in *R. nivale* than in *R. monicae*, respectively. As DGGA synthesis has been previously shown to compensate for the SQDG deficiency in *sqs1* and *ugp3* mutants lacking SQDG in *A. thaliana* (Okazaki et al., 2013), it is reasonable to postulate that DGGA in P‐starved cells of *R. monicae* increased to compensate for decrease in PG together with/instead of SQDG. Taken together, we propose that DGGA metabolically compensated for the PG deficiency. DGGA may also replace PI, taking into account their quantitatively inverse regulation under P‐starvation, that is, similarity in acyl composition and the anionic nature of their lipid classes (Okazaki et al., 2013). Generating mutants lacking DGGA would help clarify the reciprocal relationships between DGGA and other lipid classes including PI, PG as well as SQDG, and elucidate the function of DGGA in microalgae.

Microalgae have acquired diverse lipid classes as components of structural membranes and adopted various phospholipid remodeling strategies to cope with P‐limited environment. Here, our study unraveled an additional unique adaptative strategy to P‐deficient conditions in *R. monicae* and *R. nivale* where two non‐P containing polar lipids DGTS and DGGA may play pivotal roles. The use of DGGA under P‐starvation could also benefit by minimizing nitrogen requirements for algae since DGGA contains no elemental nitrogen, unlike DGTS. The ability of *Raphidonema* to replace phospholipids with both DGTS and DGGA may have benefited the colonization of this species in cold harsh environments such as snow and ice where nutrient supply could be very low and where cells simultaneously face multiple forms of stress (Ezzedine et al., 2023; McCutcheon et al., 2021). DGGA was not previously recognized in Chlorophyta, and therefore further investigation of such lipid remodeling in other organisms requires a wide range of microalgal species in different taxonomic groups or habitats for a comprehensive understanding of the strategies to P‐deficient environments.

## CONCLUSION

The present study has revealed the landscape of complex lipid molecular species and lipid remodeling dynamics under P‐starvation in unexplored cold‐adapted microalgal strains belonging to genus *Raphidonema* (Chlorophyta). We describe a new lipid remodeling pattern where lipid class DGGA and betaine lipid DGTS potentially play significant roles as phospholipid replacements in P‐limited environments. The lipidomic architecture presented in this study identifying the presence of VLC‐PUFAs and DGGA makes *Raphidonema* an attractive model to further elucidate the regulation of structural lipids, their pathways, as well as physiological roles in maintaining the functionality of biological membranes. Collectively, these findings offer a new insight into possible adaptation mechanisms of microalgae to cope with low‐P environments, while expanding our understanding of diverse lipid remodeling responses of the species of Chlorophyta with important consequences for biogeochemistry in natural environments or for bioprocessing of high value lipids for biotechnological purposes.

## EXPERIMENTAL PROCEDURES

### General cultivation conditions

*Raphidonema monicae* strain SAG 2030 (Chlorophyta, Trebouxiophyceae) was obtained from the Culture Collection of Algae at Göttingen University (SAG, Göttingen, Germany) and originally isolated from Terra Nova Bay, Ross sea in Antarctica, whilst *Raphidonema nivale* strain CCCryo 559–22 (Chlorophyta, Trebouxiophyceae) was isolated from snow in Northern Norway (Suzuki et al., 2023). Stock cultures were kept on 1.2% agar plates as single cell colonies. Cells were then grown in 320 mL tubes containing modified Bolds Basal Medium (BBM) (Bischoff and Bold 1963) sparged with air containing 1% CO_2_, unless otherwise stated. The tubes were supplied with a photosynthetic photon flux density (PPFD) of 90 ± 20 μmol⋅m^−2^⋅s^−1^ using cool white fluorescent lamps. The bioreactor vessels and nutrient media used in each set of the following experiments were sterilized by autoclave (121°C, 20 min). Two independent experiments were conducted; the first one (Experiment 1) was with *R. monicae* in turbidostat cultures to determine the effects of P starvation on growth, cellular P content, fatty acids and lipid class composition whereas in the second study (Experiment 2) both *R. monicae* and *R. nivale* were employed in batch cultures to study the effects on growth, lipid class, and lipid molecular species composition.

For the first experiments, *R. monicae* was continuously cultivated in flat‐plate photobioreactors (Algaemist, Technical Development Studio, Wageningen University, Wageningen, the Netherlands) with a working volume of 0.4 L, an optical depth of 14 mm, and an illuminated area of 0.028 m^2^. Bioreactor temperature was accurately controlled with an external cooling system (Julabo F25, JULABO GmbH, Seelbach, Germany) and an internal heating system maintaining 15°C, which is optimal temperature of the strain (Vona et al., 2004). Cultures were sparged with 0.2 μm filtered air (Acrodisc PTFE filters, Pall Corporation, New York, USA) enriched with 5.0% CO_2_. The reactors were illuminated continuously with warm white LEDs from one side at 337 ± 13 μmol⋅m^−2^⋅s^−1^ photons photosynthetically active radiation (PAR). A light sensor on the backside of reactors was used to monitor the light passing through the reactor and if the emitted light intensity dropped below the required setpoint, the culture was automatically diluted with fresh medium connected to a peristaltic pump. The turbidostat maintained cell density to ensure consistent light intensity throughout the experiment. The PPFD impinging on the front of the cultivation vessel was measured with a Li‐Cor189 2π quantum sensor (Li‐Cor, Lincoln, USA). The incident light measurements represent the average values obtained at 28 positions. Turbidostat cultures in both nutrient replete and P‐starved conditions were initially operated using 3 × 3 N‐BBM medium (three times concentrated 3 N‐BBM with Tris base buffer 1.5 g⋅L^−1^). After the cultures were grown at steady‐state for 4 days, the cells were centrifuged (980 *g*, 5 min), washed and resuspended in either nutrient replete medium (3 × 3 N BBM medium; +P) or P‐starved medium (3 × 3 N‐BBM without KH_2_PO_4_ or K_2_HPO_4_; ‐P). After washing and resuspending in the experimental media, the starting cell density of each reactor was approximately the same at 3.8 g⋅L^−1^ dry weight (DW). The timing of washing and the application of experimental treatment was designated at the start of the experiment (*t* = 0), and the subsequent cultivation time was 6 days. Each treatment (+P and –P) was performed with four replicates. The biomass concentration and the bioreactor outflow volume were measured daily, and samples for analysis were collected daily. The growth rate (d^−1^) was calculated by dividing the outflow of the reactor (L⋅d^−1^) by the reactor volume (0.4 L) based on the assumption that cell density was constant throughout the experiments. The medium was continuously mixed with magnetic stirring bars.

For the second experiments, *R. monicae* and *R. nivale* were initially cultivated in 300 mL of 3 × 3 N‐BBM (control) in bubble tubes in batch cultures, illuminated with 100 ± 5 μmol photons⋅m^−2^⋅s^−1^ PAR, sparged with 0.2 μm filtered air enriched with 1% CO_2_ in a climate chamber maintained at 10 °C. *R. nivale* strain grew strongly at 10 °C in the previous study (Suzuki et al., 2023), and this temperature was chosen to ensure comparable growth between the two strains. When the cultures reached the exponential phase (optical density of 1.0 at 540 nm), the cells were centrifuged (3500 *g*, 2 min), washed, and resuspended in one of two treatments; the control (C) or P‐starved (‐P) medium as employed in the first experiments (*n =* 4). The time of washing and the application of experimental treatments was designated at the start of the experiment (*t* = 0), and the subsequent cultivation time was 6 days. Each treatment was performed in four biological replicates and randomly assigned to one of the bubble‐tubes. Cells were harvested by centrifugation at 4500 ***g*** for 2 min and immediately frozen in liquid nitrogen and stored at −80°C.

### Photosynthetic measurements

Maximum quantum yield of photosystem II (Fv/Fm) was measured with a Multi‐Color PAM chlorophyll fluorometer (Heinz Walz GmbH, Pfullingen, Germany). Cells were dark acclimated for 20 min prior to the experiments, and the maximum fluorescence signal (*F*_*m*_) and fluorescence baseline (*F*_*0*_) were obtained (*n* = 4) by the saturation pulse. Maximum quantum yield of photosystem II (*F*_*v*_/*F*_*m*_) was calculated according to the equation:

$$\frac{F_{m}-F_{0}}{F_{m}}=\frac{F_{v}}{F_{m}},$$

where *F*_*0*_ is the minimal fluorescence under modulated measuring light, *F*_*m*_ is the maximum fluorescence under saturating light.

### Cellular and extracellular phosphorus content

Persulfate‐oxidized freeze‐dried cell samples were used for analysis of intracellular P content, where 2.5 mg of dried biomass was digested in a mixture of 50 mL of distilled water and 5 mL of the oxidizing reagent in an autoclave for 30 mins, as described by Grasshoff et al. (2009). The supernatant of the culture broth was used for extracellular P content. The digested cellular and extracellular P contents were each quantified with the colorimetric ascorbic acid/molybdate method (Ringuet et al., 2011). The absorbance of the colored complex was measured at 880 nm and calibrated with five‐point standard curves of KH_2_PO_4_.

### Lipid extraction

In the first experiments, total lipids were extracted from approximately 5 mg of freeze‐dried biomass using 4.0 mL of chloroform: methanol (2:1 v/v). A bead mill (Precellys, Bertin Technologies, Montigny‐le‐Bretonneux, France) and 150–212 μm glass beads (Sigma, St. Louis, USA) were used for lysing the cells at 4°C. Lipids were recovered by addition of 2.5 mL Tris buffer (mM Tris, NaCl, pH 7.5) followed by sonication for 10 min, mix by a vortex (10s) and centrifugation (3000 *g*, 5 min). The chloroform phase containing the crude lipid extract was removed and dried under a stream of N_2_. In the second experiments, known volume of frozen cells were gently resuspended with hot methanol containing 0.01% butylated hydroxytoluene (w/v) for 10 min at 85°C. Samples are then vortexed for 5 min to break the cells. Methyl tert‐butyl ether (MTBE) was added to reach the ratio of MTBE: methanol (5:1) followed by a vortex. Aqueous solution (NaCl 0.9%) was added and centrifuged (3000 ***g***, 5 min) with the final ratio of MTBE/methanol/water (NaCl 0.9%) (5/1/1, v/v/v) to allow phase separation. MTBE phase containing the crude lipid extract was transferred to a new glass tube. A mixture of MTBE/methanol/water (NaCl 0.9%) (1/1/1, v/v/v) was used to re‐extract the lipids in the original tube. MTBE phase was again added to the new glass tube and dried under a stream of N_2_. The extracted lipids were resuspended into chloroform and methanol mix (2:1, v/v) or acetonitrile/isopropanol/ammonium acetate 10 mM mix (65:30:5, v/v/v) for TLC analysis and UPLC‐MS/MS: ultra‐performance liquid chromatography (RS 3000, Thermo Fisher, Waltham, MA, USA) connected to a QTOF 5600 mass spectrometer (AB Sciex, Framingham, MA, USA), respectively.

### Lipid class quantification by high performance thin layer chromatography (HPTLC) and lipid molecular species analysis by UPLC‐MS/MS

Lipid classes were separated by HPTLC and quantified by densiometry with a standard calibration curve. Ten μL of total extracted lipids was loaded onto a 10 × 20 cm silica gel 60 F254 TLC plate (Merck, Darmstadt, Germany) using an ATS5 automatic TLC sampler (Camag, Muttenz, Switzerland). In the first experiments, the TLC plates were developed by either by a solvent mix of hexane: diethlyether: acetic acid (17/3/0.2, v/v/v) for neutral lipids or by a mix of chloroform:acetone:methanol:acetic acid:water 25% (50:20:10:10:5, v/v/v/v) for polar lipid separation. Phospholipids included PE, PG, and PI whilst polar lipids without phosphorus contained MGDG, DGDG, SQDG, DGTS and DGGA. In the second experiments, the TLC plate was developed by a mixture of hexane/diethyl ether/acetic acid (35/15/0.5, v/v/v) for neutral lipid separation or a mixture of chloroform/acetone/methanol/acetic acid/water (25/10/7.5/6/4, v/v/v/v) for polar lipid separation. TLC plates were then dried and dipped for 6 sec in a staining reagent (2% CuSO_4_, 8% H_3_PO_4_ in water), followed by heating at 180°C for 30 min (neutral lipid) and at 172°C for 20 min (polar lipid) on a TLC plate heater. Subsequently, the plates were scanned by a TLC Scanner 3 with WinCATs software (Camag, Muttenz, Switzerland). The lipid standards used were MGDG, DGDG, SQDG, PC, PG, phosphatidic acid, phosphatidylserine, and phosphatidylethanolamine (PE) from Avanti Polar Lipids, Inc. (Alabaster, AL, USA).

To identify an unknown lipid class DGGA in UPLC‐MS/MS, these lipids were first separated from the other lipid classes on another plate by two‐dimensional TLC using TLC silica plates (Merck, Darmstadt, Germany); the solvent system employed for the first‐dimension run was chloroform:methanol:water:ammonia 25% (30/17/2.75/0.25, v/v/v/v) while for the second dimension a mix of chloroform:acetone:methanol:acetic acid:water (50/20/10/10/5, v/v/v/v/v) was used. Each lipid class spot on TLC plates was visualized by spray with 0.01% (w/v) primulin in 80% (v/v) acetone, irradiated with UV light (254 nm), and gently marked by pencil. It was then scraped off from the TLC plate and resuspended with adding 0.5 mL of chloroform and methanol (2/1, v/v) in a glass tube. Then it was mixed with a vortex and the solvent containing lipid was transferred through a glass Pasteur pipette stiffed with a pinch of glass wool at the bottom to remove silica. The solvents were then dried under N_2_ gas, resuspended into 200 μL of a mixture of acetonitrile/isopropanol/ammonium acetate 10 mM (65/30/5, v/v/v) and the lipid samples were readied for mass spectrometric analysis. Lipid classes or the molecular species of the individual lipid class was identified and quantified by UPLC‐MS/MS as described by Légeret et al. (2016) and Nguyen et al. (2013).

### Genome sequencing, assembly, annotation of *R. Monicae*

*Raphidonema monicae* was cultivated in a 400 mL photobioreactor in turbidostat mode. Cells were collected and pelleted by centrifugation, then the DNA was immediately extracted using an E.Z.N.A. Plant extraction kit (Omega Biotek, Georgia, USA). The DNA sample was sent to NovoGene (Hong Kong) for whole‐genome sequencing. DNA libraries were sequenced with Illumina using 150 bp paired end reads, yielding 13.2 GB of data that was subject to adapter trimming and quality control. Sequence reads were assembled with SOAPdenovo2, then the assembly was curated for contamination and genes structurally annotated by NovoGene. The *R. monicae* genome assembly was 55.358 MB in size. As a draft, short read only assembly, the genome consisted of 10 928 contigs with N50 1707 KB and L50 9.281 KB. The GC content was 55.57%, with 10 735 annotated coding sequences. The assembly size is supported with k‐mer frequency analysis using the 19‐mer profile computed by Jellyfish v.2.2.6, which estimated a genome of 63.9 Mb (Fig. S1). BUSCO analysis of the genome sequence was conducted using the chlorophyta_odb10 reference set of 1519 single copy orthologs using—augustus and—long optimization identified 72.9% complete, 0.7% duplicated, 11.7% fragmented, and 15.4% missing genes (Table S1). The genome sequence, GFF annotation, and functional annotation files are hosted at Zenodo under https://doi.org/10.5281/zenodo.11506421.

The peptide amino‐acid sequences were functionally annotated using three complimentary methods. The protein sequences were first annotated by blastp against the SwissProt database (Boutet et al., 2007), then ontology terms identified with InterProScan (Jones et al., 2014) and Emapper.py (Huerta‐Cepas et al., 2017), and the results were combined and curated in Blast2GO. The protein sequences from the whole genome annotation (10 735 amino acid sequences) were built into a blast database using Geneious Prime version 2022.2.2. As SQDG synthase are known to be involved in the DGGA synthesis in the higher plant *Arabidopsis thaliana* (Okazaki et al., 2013), 273 SQDG synthase protein sequences reported in microalgae from the study of Riccio et al. (2020) were compared with the database of *R. monicae*. Two putative SQDG synthase genes were identified within the draft genome of *R. monicae*, which were KAD1A03384 and KAD1A10511.

### Phylogenetic analysis

The protein sequences of putative SQDG synthase from algae and plants were retrieved from available data including NCBI and Phytozome (Goodstein et al., 2012), were aligned and trimmed by MEGAX 10.1.8 (Kumar et al., 2018) to obtain the same length. The total of 326 aligned protein sequences were used for the construction of phylogenetic tree using the maximum‐likelihood method with the amino acid substitution model of LG model + Gamma, which was selected as the best model based on the Bayesian information criterion calculated by the same software. The bootstrapping values (1000 replicates) and Bayesian posterior probabilities were calculated by MEGAX 10.1.8 and MrBayes 3.2.6 (Ronquist & Huelsenbeck, 2003), respectively.

## Author Contributions

HS conceived and designed the research under the supervision of RW, CJH, YL, and VK. HS performed cultivation experiments and its associated analyses. HS and SC conducted lipid analyses. BL conducted UPLC‐MS/MS analysis and extracted the relevant data for the MS. HS conducted genome and phylogenetic analyses with help of CJH. HS analyzed the data and wrote the manuscript. All authors read and contributed to the final version of the manuscript.

## Conflict of Interests

There are no conflicts of interest.

## Supporting information


**Figure S1.** The K‐mer frequency distribution analysis of whole‐genome Illumina reads and genome size estimation of *Raphidonema monicae*.
**Figure S2.** A chromatogram of lipids extracted from *Raphidonema monicae* in control (pink line) and P‐starved (‐P; blue line) conditions from day 6 of a batch culture experiment analyzed by ultra‐performance liquid chromatography connected to a QTOF 5600 mass spectrometer. The number next to the peaks represent the retention time (minutes).
**Figure S3.** A chromatogram of lipids extracted from *Raphidonema nivale* in control (blue line) and P‐starved (‐P; pink line) conditions from day 6 of a batch culture experiment analyzed by ultra‐performance liquid chromatography equipped with a QTOF 5600 mass spectrometer. The number next to the peaks represent the retention time (minutes).
**Figure S4.** The dynamics of lipid molecular species in chloroplast lipid classes in *Raphidonema monicae* at day 1, 3, 5, and 6 in control (non‐P starved) and P‐starved conditions in a batch culture experiment. Data represent the mean ± standard deviation of four biological replicates. MGDG, monogalactosyldiacylglycerol; DGDG, digalactosyldiacylglycerol; SQDG, sulfoquinovosyldiacylglycerol; PG, phosphatidylglycerol.
**Figure S5.** The dynamics of lipid molecular species in extra‐chloroplast lipid classes and DGGA in *Raphidonema monicae* at day 1, 3, 5, and 6 in control (non‐P starved) and P starved batch cultures. Data represent the mean ± standard deviation of four biological replicates. DGGA, diacylglyceryl glucuronide; DGTS, 1,2‐diacylglyceryl‐3‐*O*‐4′‐(*N,N,N*‐trimethyl)‐homoserine; PI, phosphatidylinositol; PE, phosphatidylethanolamine; PC, phosphatidylcholine.
**Table S1.** BUSCO analysis results for evaluating the completeness of the genome annotation of *Raphidonema monicae*.
**Table S2.** Glycerolipid species identified in *Raphidonema monicae* analyzed by ultra‐performance liquid chromatography equipped with a QTOF 5600 mass spectrometer. FA, fatty acid.

## Data Availability

The data that support the findings of this study are openly available in Zenodo at http://doi.org/10.5281/zenodo.11506421.
