# Supplementary material for: Phosphorus starvation induces the synthesis of novel lipid class diacylglyceryl glucuronide and diacylglyceryl‐N,N,N‐trimethylhomoserine in two species of cold‐adapted microalgae Raphidonema (Chlorophyta)
Source: Plant J. 2025 Jan 27;121(2):e17227. doi: 10.1111/tpj.17227 (PMC11771548; doi:10.1111/tpj.17227)
Supplement: Supplementary file 1 — Figure S1. The K‐mer frequency distribution analysis of whole‐genome Illumina reads and genome size estimation of Raphidonema monicae. Figure S2. A chromatogram of lipids extracted from Raphidonema monicae in control (pink line) and P‐starved (‐P; blue line) conditions from day 6 of a batch culture experiment analyzed by ultra‐performance liquid chromatography connected to a QTOF 5600 mass spectrometer. The number next to the peaks represent the retention time (minutes). Figure S3. A chromatogram of lipids extracted from Raphidonema nivale in control (blue line) and P‐starved (‐P; pink line) conditions from day 6 of a batch culture experiment analyzed by ultra‐performance liquid chromatography equipped with a QTOF 5600 mass spectrometer. The number next to the peaks represent the retention time (minutes). Figure S4. The dynamics of lipid molecular species in chloroplast lipid classes in Raphidonema monicae at day 1, 3, 5, and 6 in control (non‐P starved) and P‐starved conditions in a batch culture experiment. Data represent the mean ± standard deviation of four biological replicates. MGDG, monogalactosyldiacylglycerol; DGDG, digalactosyldiacylglycerol; SQDG, sulfoquinovosyldiacylglycerol; PG, phosphatidylglycerol. Figure S5. The dynamics of lipid molecular species in extra‐chloroplast lipid classes and DGGA in Raphidonema monicae at day 1, 3, 5, and 6 in control (non‐P starved) and P starved batch cultures. Data represent the mean ± standard deviation of four biological replicates. DGGA, diacylglyceryl glucuronide; DGTS, 1,2‐diacylglyceryl‐3‐O‐4′‐(N,N,N‐trimethyl)‐homoserine; PI, phosphatidylinositol; PE, phosphatidylethanolamine; PC, phosphatidylcholine. Table S1. BUSCO analysis results for evaluating the completeness of the genome annotation of Raphidonema monicae. Table S2. Glycerolipid species identified in Raphidonema monicae analyzed by ultra‐performance liquid chromatography equipped with a QTOF 5600 mass spectrometer. FA, fatty acid. [file TPJ-121-0-s001.docx]

# Supplementary information

## Supporting Figures:


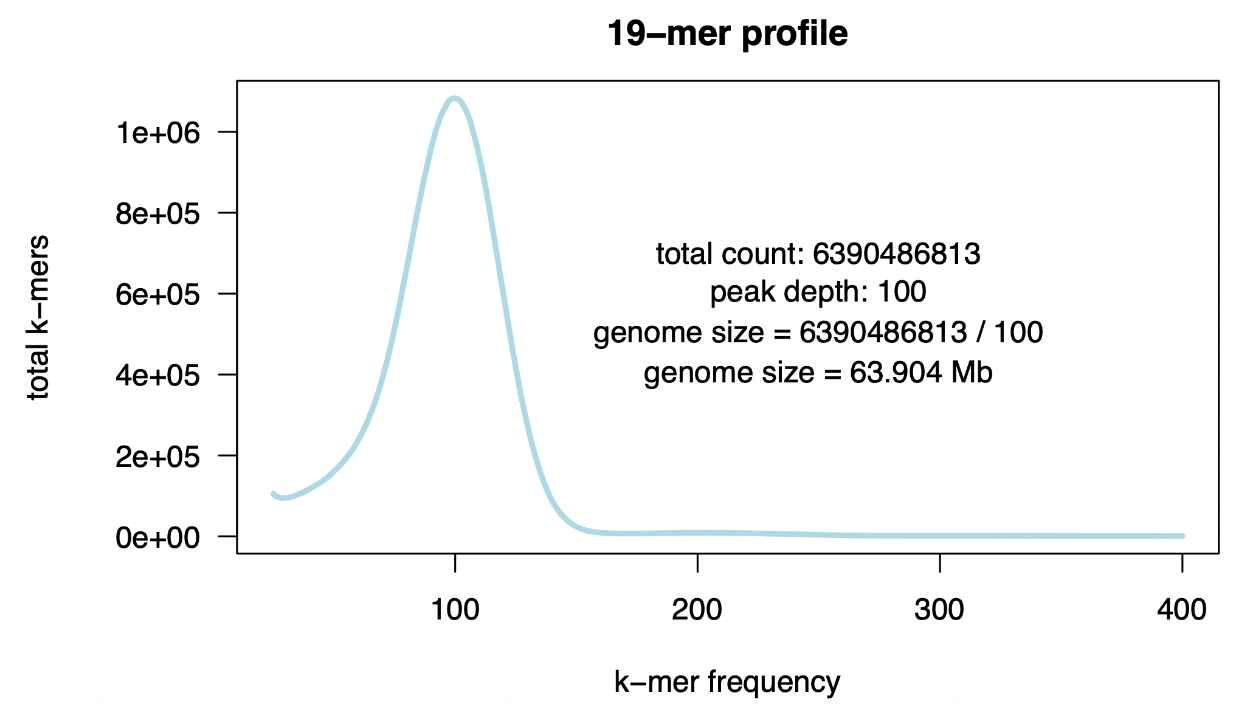


**Fig. S1.** The K-mer frequency distribution analysis of whole-genome Illumina reads and genome size estimation of *Raphidonema monicae.*


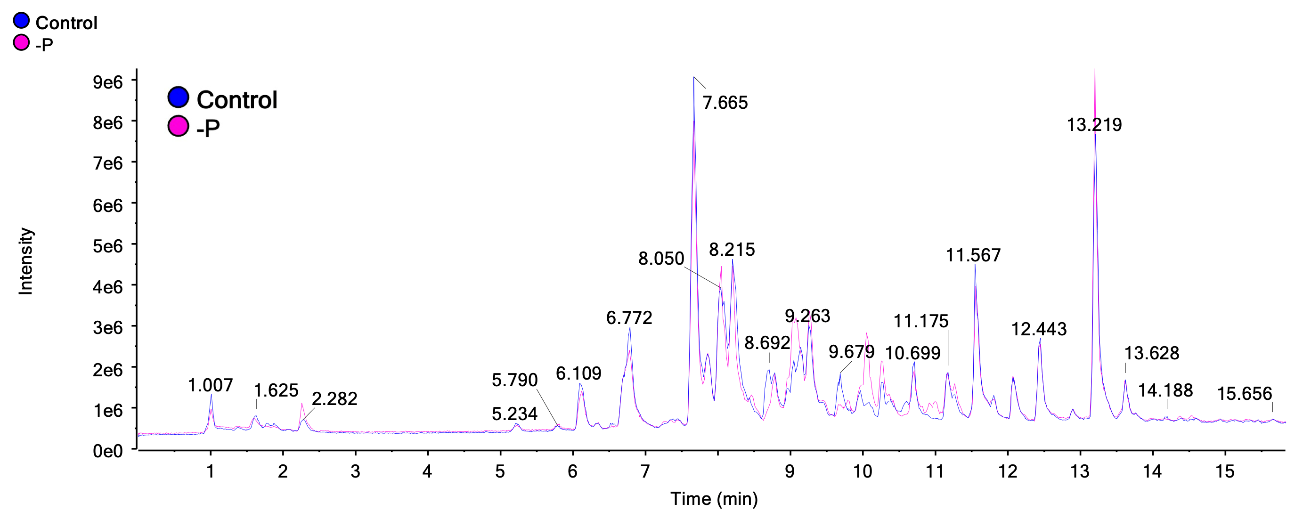


**Fig. S2.** A chromatogram of lipids extracted from *Raphidonema monicae* in control (pink line) and P-starved (-P;blue line) conditions from day 6 of a batch culture experiment analyzed by ultra-performance liquid chromatography connected to a QTOF 5600 mass spectrometer. The number next to the peaks represent the retention time (minutes).


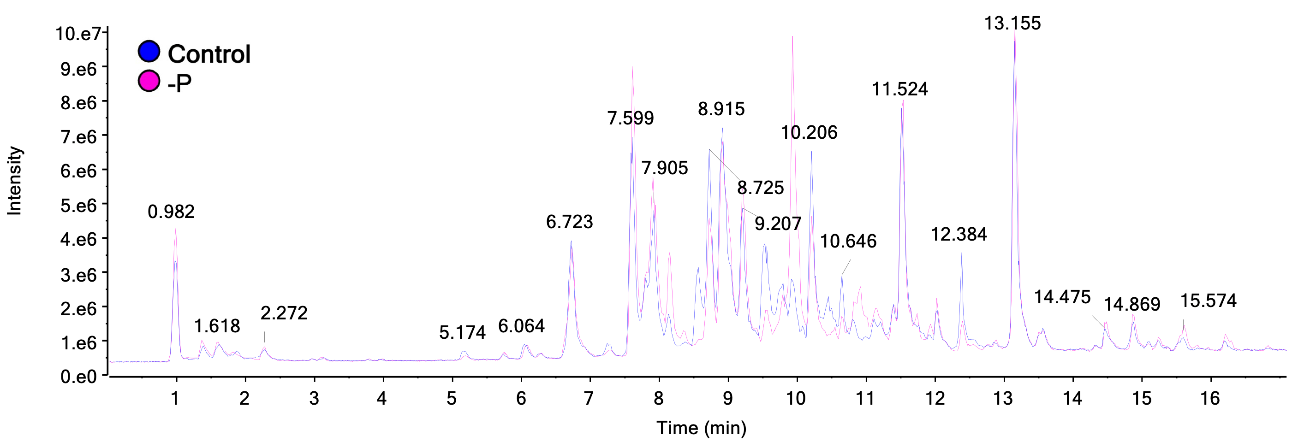


**Fig. S3.** A chromatogram of lipids extracted from *Raphidonema nivale* in control (blue line) and P-starved (-P; pink line) conditions from day 6 of a batch culture experiment analyzed by ultra-performance liquid chromatography equipped with a QTOF 5600 mass spectrometer. The number next to the peaks represent the retention time (minutes).

**Fig. S4**. The dynamics of lipid molecular species in chloroplast lipid classes in *Raphidonema monicae* at day 1, 3, 5, and 6 in control (non-P starved) and P-starved conditions in a batch culture experiment. Data represent the mean ± standard deviation of four biological replicates. MGDG, monogalactosyldiacylglycerol; DGDG, digalactosyldiacylglycerol; SQDG, sulfoquinovosyldiacylglycerol; PG, phosphatidylglycerol.

** Fig. S5**. The dynamics of lipid molecular species in extra-chloroplast lipid classes and DGGA in *Raphidonema monicae* at day 1, 3, 5, and 6 in control (non-P starved) and P starved batch cultures. Data represent the mean ± standard deviation of four biological replicates. DGGA, diacylglyceryl glucuronide; DGTS, 1,2-diacylglyceryl-3-*O*-4’-(*N,N,N*-trimethyl)-homoserine; PI, phosphatidylinositol; PE, phosphatidylethanolamine; PC, phosphatidylcholine.

## Supporting tables:

**Table S1.** BUSCO analysis results for evaluating the completeness of the genome annotation of *Raphidonema monicae*.

| **Results from dataset chlorophyta_odb 10** |
| --- |
| C:65.9%[S:64.3%, D:1.6%], F:9.9%, M:24.2%, n:1519 |
| 1001 Complete BUSCOs (C) |
| 977 Complete and single-copy BUSCOs (S) |
| 24 Complete and duplicated BUSCOs (D) |
| 151 Fragmented BUSCOs (F) |
| 367 Missing BUSCOs (M) |
| 1519 Total BUSCO groups searched |

**Table S2.** Glycerolipid species identified in *Raphidonema* *monicae* analyzed by ultra-performance liquid chromatography equipped with a QTOF 5600 mass spectrometer. FA, fatty acid.

| Lipid class | Formula | FA1 | FA2 | Adduct | Mass |
| --- | --- | --- | --- | --- | --- |
| **MGDG** |  |  |  |  |  |
| MGDG 34:1 | C_43_H_80_O_10_ | 16:0 | 18:1 | HCOO | 801.6 |
| MGDG 34:2 | C_43_H_78_O_10_ | 16:1 | 18:1 | HCOO | 799.6 |
| MGDG 34:2 | C_43_H_78_O_10_ | 16:0 | 18:2 | HCOO | 799.6 |
| MGDG 34:3 | C_43_H_76_O_10_ | 16:0 | 18:3 | HCOO | 797.5 |
| MGDG 34:3 | C_43_H_76_O_10_ | 16:1 | 18:2 | HCOO | 797.5 |
| MGDG 34:4 | C_43_H_74_O_10_ | 16:2 | 18:2 | HCOO | 795.5 |
| MGDG 34:4 | C_43_H_74_O_10_ | 16:3 | 18:1 | HCOO | 795.5 |
| MGDG 34:4 | C_43_H_74_O_10_ | 16:1 | 18:3 | HCOO | 795.5 |
| MGDG 34:5 | C_43_H_72_O_10_ | 16:2 | 18:3 | HCOO | 793.5 |
| MGDG 34:5 | C_43_H_72_O_10_ | 16:3 | 18:2 | HCOO | 793.5 |
| MGDG 34:6 | C_43_H_70_O_10_ | 16:3 | 18:3 | HCOO | 791.5 |
| MGDG 34:6 | C_43_H_70_O_10_ | 16:4 | 18:2 | HCOO | 791.5 |
| MGDG 34:7 | C_43_H_68_O_10_ | 16:4 | 18:3 | HCOO | 789.5 |
| MGDG 34:8 | C_43_H_66_O_10_ | 16:4 | 18:4 | HCOO | 787.5 |
| MGDG 36:3 | C_45_H_80_O_10_ | 18:1 | 18:2 | HCOO | 825.6 |
| **DGDG** |  |  |  |  |  |
| DGDG 34:2 | C_49_H_88_O_15_ | 16:1 | 18:1 | HCOO | 961.6 |
| DGDG 34:3 | C_49_H_84_O_15_ | 16:0 | 18:3 | HCOO | 959.6 |
| DGDG 34:3 | C_49_H_84_O_15_ | 16:1 | 18:2 | HCOO | 959.6 |
| DGDG 34:4 | C_49_H_84_O_15_ | 16:2 | 18:2 | HCOO | 957.6 |
| DGDG 34:5 | C_49_H_82_O_15_ | 16:2 | 18:3 | HCOO | 955.6 |
| DGDG 34:5 | C_49_H_82_O_15_ | 16:3 | 18:2 | HCOO | 955.6 |
| DGDG 34:6 | C_49_H_80_O_15_ | 16:3 | 18:3 | HCOO | 953.5 |
| DGDG 34:7 | C_49_H_78_O_15_ | 16:4 | 18:3 | HCOO | 951.5 |
| DGDG 34:8 | C_49_H_76_O_15_ | 16:4 | 18:4 | HCOO | 949.5 |

| **SQDG** |  |  |  |  |  |
| --- | --- | --- | --- | --- | --- |
| SQDG 32:0 | C_41_H_78_O_12_S | 16:0 | 16:0 | -H | 793.5 |
| SQDG 34:1 | C_43_H_76_O_12_S | 16:0 | 18:1 | -H | 819.5 |
| SQDG 34:2 | C_43_H_78_O_12_S | 16:0 | 18:2 | -H | 817.5 |
| SQDG 34:3 | C_43_H_78_O_12_S | 16:0 | 18:3 | -H | 815.5 |
| SQDG 36:6 | C_45_H_74_O_12_S | 18:3 | 18:3 | -H | 837.5 |
| **PG** |  |  |  |  |  |
| PG 30:0 | C_38_H_75_O_10_P | 14:0 | 16:0 | -H | 693.5 |
| PG 32:0 | C_38_H_75_O_10_P | 16:0 | 16:0 | -H | 721.5 |
| PG 32:1 | C_38_H_73_O_10_P | 18:1 | 14:0 | -H | 719.5 |
| PG 32:1 | C_38_H_73_O_10_P | 16:0 | 16:1 | -H | 719.5 |
| PG 34:1 | C_40_H_77_O_10_P | 14:0 | 20:1 | -H | 747.5 |
| PG 34:1 | C_40_H_77_O_10_P | 16:0 | 18:1 | -H | 747.5 |
| PG 34:2 | C_40_H_75_O_10_P | 16:0 | 18:2 | -H | 745.5 |
| PG 34:3 | C_40_H_73_O_10_P | 16:0 | 18:3 | -H | 743.5 |
| PG 34:3 | C_40_H_73_O_10_P | 16:1 | 18:2 | -H | 743.5 |
| PG 34:4 | C_40_H_71_O_10_P | 16:1 | 18:3 | -H | 741.5 |
| PG 36:1 | C_42_H_81_O_10_P | 16:0 | 20:1 | -H | 775.5 |
| **PI** |  |  |  |  |  |
| PI 32:2 | C_41_H_75_O_13_P | 14:0 | 18:2 | -H | 805.5 |
| PI 34:1 | C_43_H_79_O_13_P | 16:0 | 18:1 | -H | 835.5 |
| PI 34:2 | C_43_H_79_O_13_P | 16:0 | 18:2 | -H | 833.5 |
| PI 34:3 | C_43_H_77_O_13_P | 16:0 | 18:3 | -H | 831.5 |
| PI 34:4 | C_43_H_75_O_13_P | 16:0 | 18:4 | -H | 829.5 |
| PI 36:5 | C_43_H_75_O_13_P | 18:2 | 18:3 | -H | 855.5 |
| **PE** |  |  |  |  |  |
| PE 38:4 | C_43_H_78_O_8_PN | 18:1 | 20:3 | -H | 766.5 |
| PE 38:5 | C_43_H_76_O_8_PN | 18:1 | 20:4 | -H | 764.5 |
| PE 38:6 | C_43_H_74_O_8_PN | 18:1 | 20:5 | -H | 762.5 |
| PE 40:10 | C_45_H_70_O_8_PN | 20:5 | 20:5 | -H | 782.5 |
| PE 40:5 | C_45_H_80_O8PN | 20:1 | 20:4 | -H | 792.6 |
| PE 40:6 | C_45_H_70_O_8_PN | 20:1 | 20:5 | -H | 790.5 |
| PE 40:8 | C_45_H_74_O_8_PN | 20:3 | 20:5 | -H | 788.5 |
| PE 40:8 | C_45_H_74_O_8_PN | 20:4 | 20:4 | -H | 788.5 |
| PE 40:9 | C_45_H_72_O_8_PN | 20:4 | 20:5 | -H | 786.5 |
| PE 44:5 | C_49_H_88_O_8_PN | 20:5 | 24:0 | -H | 848.6 |
| PE 44:6 | C_49_H_86_O_8_PN | 20:5 | 24:1 | -H | 846.6 |
| **PC** |  |  |  |  |  |
| PC 34:2 | C_42_H_80_O_8_PN | 16:0 | 18:2 | HCOO | 802.6 |
| PC 34:3 | C_42_H_78_O_8_PN | 16:0 | 18:3 | HCOO | 800.5 |
| PC 34:3 | C_42_H_78_O_8_PN | 18:3 | 16:0 | HCOO | 800.5 |
| PC 34:4 | C_42_H_76_O_8_PN | 16:0 | 18:4 | HCOO | 798.5 |
| PC 34:7 | C_42_H_70_O_8_PN | 16:4 | 18:3 | HCOO | 792.5 |
| PC 36:4 | C_44_H_80_O_8_PN | 18:3 | 18:1 | HCOO | 826.6 |
| PC 36:4 | C_44_H_80_O_8_PN | 16:0 | 20:4 | HCOO | 826.6 |
| PC 36:5 | C_44_H_78_O_8_PN | 18:4 | 18:1 | HCOO | 779.5 |
| PC 36:5 | C_44_H_78_O_8_PN | 18:3 | 18:3 | HCOO | 779.5 |
| PC 38:5 | C_46_H_82_O_8_PN | 20:4 | 18:1 | HCOO | 852.6 |
| PC 38:5 | C_46_H_82_O_8_PN | 18:2 | 20:3 | HCOO | 852.6 |
| PC 38:7 | C_46_H_78_O_8_PN | 18:2 | 20:5 | HCOO | 848.5 |
| PC 38:7 | C_46_H_78_O_8_PN | 18:3 | 20:4 | HCOO | 848.5 |
| PC 38:8 | C_46_H_76_O_8_PN | 18:3 | 20:5 | HCOO | 846.5 |
| PC 38:8 | C_46_H_76_O_8_PN | 18:4 | 20:4 | HCOO | 846.5 |
| PC 40:10 | C_48_H_76_NO_8_P | 20:5 | 20:5 | HCOO | 870.5 |
| PC 40:9 | C_48_H_78_NO_8_P | 20:4 | 20:5 | HCOO | 872.5 |
| **DGTS** |  |  |  |  |  |
| DGTS 32:3 | C_42_H_73_O_7_N | 16:0 | 16:3 | HCOO | 748.5 |
| DGTS 32:3 | C_42_H_73_O_7_N | 14:0 | 18:3 | HCOO | 748.5 |
| DGTS 32:4 | C_42_H_73_O_7_N | 14:0 | 18:4 | HCOO | 748.5 |
| DGTS 32:4 | C_42_H_73_O_7_N | 16:0 | 16:4 | HCOO | 748.5 |
| DGTS 34:1 | C_44_H_83_O_7_N | 16:0 | 18:1 | HCOO | 782.6 |
| DGTS 34:2 | C_44_H_81_O_7_N | 16:0 | 18:2 | HCOO | 780.6 |
| DGTS 34:3 | C_44_H_79_O_7_N | 16:0 | 18:3 | HCOO | 778.6 |
| DGTS 34:4 | C_44_H_77_O_7_N | 16:0 | 18:4 | HCOO | 776.6 |
| DGTS 36:3 | C_46_H_83_O_7_N | 16:0 | 20:3 | HCOO | 806.6 |
| DGTS 36:3 | C_46_H_83_O_7_N | 18:1 | 18:2 | HCOO | 806.6 |
| DGTS 36:4 | C_46_H_81_O_7_N | 18:2 | 18:2 | HCOO | 804.6 |
| DGTS 36:4 | C_46_H_81_O_7_N | 20:4 | 16:0 | HCOO | 804.6 |
| DGTS 36:4 | C_46_H_81_O_7_N | 18:1 | 18:3 | HCOO | 804.6 |
| DGTS 36:5 | C_46_H_79_O_7_N | 18:2 | 18:3 | HCOO | 802.6 |
| DGTS 36:5 | C_46_H_79_O_7_N | 16:0 | 20:5 | HCOO | 802.6 |
| DGTS 38:4 | C_48_H_85_O_7_N | 18:3 | 20:1 | HCOO | 832.6 |
| DGTS 38:5 | C_48_H_83_O_7_N | 18:2 | 20:3 | HCOO | 830.6 |
| DGTS 38:5 | C_48_H_83_O_7_N | 18:4 | 20:1 | HCOO | 830.6 |
| DGTS 38:5 | C_48_H_83_O_7_N | 18:1 | 20:4 | HCOO | 830.6 |
| DGTS 38:7 | C_48_H_79_O_7_N | 18:2 | 20:3 | HCOO | 826.6 |
| DGTS 38:7 | C_48_H_79_O_7_N | 18:2 | 20:3 | HCOO | 826.6 |
| DGTS 38:8 | C_48_H_77_O_7_N | 18:3 | 20:5 | HCOO | 824.6 |
| **DGGA** |  |  |  |  |  |
| DGGA 34:1 | C_43_H_78_O_11_ | 16:0 | 18:1 | -H | 769.5 |
| DGGA 34:2 | C_43_H_76_O_11_ | 16:0 | 18:2 | -H | 767.5 |
| DGGA 34:3 | C_43_H_74_O_11_ | 16:0 | 18:3 | -H | 765.5 |
| DGGA 36:1 | C_45_H_80_O_11_ | 16:0 | 20:1 | -H | 797.6 |
| DGGA 36:2 | C_45_H_80_O_11_ | 16:0 | 20:2 | -H | 795.6 |
